# Dysbiosis of Salivary Microbiota in Inflammatory Bowel Disease and Its Association With Oral Immunological Biomarkers

**DOI:** 10.1093/dnares/dst037

**Published:** 2013-09-07

**Authors:** Heba S. Said, Wataru Suda, Shigeki Nakagome, Hiroshi Chinen, Kenshiro Oshima, Sangwan Kim, Ryosuke Kimura, Atsushi Iraha, Hajime Ishida, Jiro Fujita, Shuhei Mano, Hidetoshi Morita, Taeko Dohi, Hiroki Oota, Masahira Hattori

**Affiliations:** 1 Department of Computational Biology, Graduate School of Frontier Sciences, The University of Tokyo, Kashiwanoha 5-1-5, Kashiwa, Chiba 277-8561, Japan; 2 Risk Analysis Research Center, The Institute of Statistical Mathematics, 10-3, Midori-cho, Tachikawa, Tokyo 190-8562, Japan; 3 University Hospital, Faculty of Medicine, University of the Ryukyus, Uehara 207, Nishihara, Okinawa 903-0215, Japan; 4 Department of Human Biology and Anatomy, Graduate School of Medicine, University of the Ryukyus, Uehara 207, Nishihara, Okinawa 903-0215, Japan; 5 Department of Infectious, Respiratory, and Digestive Medicine, Control and Prevention of Infectious Diseases, Graduate School of Medicine, University of the Ryukyus, Uehara 207, Nishihara, Okinawa 903-0215, Japan; 6 School of Veterinary Medicine, Azabu University, Fuchinobe 1-17-71, Chuo-ku, Sagamihara, Kanagawa 252-5201, Japan; 7 Department of Gastroenterology, Research Center for Hepatitis and Immunology, Research Institute, National Center for Global Health and Medicine, Kohnodai 1-7-1, Ichikawa, Chiba 272-8516, Japan; 8 Laboratory of Genome Anthropology, Department of Anatomy, Kitasato University School of Medicine, Kitasato 1-15-1, Minami-ku, Sagamihara, Kanagawa 252-0674, Japan

**Keywords:** Crohn's disease, ulcerative colitis, salivary microbiota, 16S rRNA, pyrosequencing

## Abstract

Analysis of microbiota in various biological and environmental samples under a variety of conditions has recently become more practical due to remarkable advances in next-generation sequencing. Changes leading to specific biological states including some of the more complex diseases can now be characterized with relative ease. It is known that gut microbiota is involved in the pathogenesis of inflammatory bowel disease (IBD), mainly Crohn's disease and ulcerative colitis, exhibiting symptoms in the gastrointestinal tract. Recent studies also showed increased frequency of oral manifestations among IBD patients, indicating aberrations in the oral microbiota. Based on these observations, we analyzed the composition of salivary microbiota of 35 IBD patients by 454 pyrosequencing of the bacterial 16S rRNA gene and compared it with that of 24 healthy controls (HCs). The results showed that Bacteroidetes was significantly increased with a concurrent decrease in Proteobacteria in the salivary microbiota of IBD patients. The dominant genera, *Streptococcus*, *Prevotella*, *Neisseria*, *Haemophilus*, *Veillonella*, and *Gemella*, were found to largely contribute to dysbiosis (dysbacteriosis) observed in the salivary microbiota of IBD patients. Analysis of immunological biomarkers in the saliva of IBD patients showed elevated levels of many inflammatory cytokines and immunoglobulin A, and a lower lysozyme level. A strong correlation was shown between lysozyme and IL-1β levels and the relative abundance of *Streptococcus*, *Prevotella*, *Haemophilus* and *Veillonella*. Our data demonstrate that dysbiosis of salivary microbiota is associated with inflammatory responses in IBD patients, suggesting that it is possibly linked to dysbiosis of their gut microbiota.

## 1. Introduction

Current advances of next-generation sequencing technologies (NGS) have enabled us to acquire massive DNA sequence data from any types of samples.^1^ In particular, complex bacterial communities composed of numerous species in various environments including human body has become the practically feasible targets, and the analysis has been shifting to the DNA-based approach in conjugation with bioinformatics for enumerated data of metagenome and 16S rRNA gene (16S) produced by NGS.^2–5^ Among these approaches, pyrosequencing-based 16S gene analysis is rapid and cost effective to comprehensively evaluate the overall structure of bacterial communities and to identify species present in them, irrespective of the yet-uncultured species.^6^ This method includes targeted PCR amplification of 16S rRNA gene variable regions with appropriate primers, followed by sequencing of the 16S amplicons using 454 pyrosequencer.^7–10^ We recently developed the improved analytical pipeline for pyrosequencing data of 16S rRNA gene V1–V2 variable region for human gut microbiota, by reassessing a PCR primer sequence, clustering conditions of error-prone 16S reads, and the quality check process to effectively remove low-quality data, and thereby the pipeline provided the high quantitative accuracy to estimation of the bacterial composition and abundance in the community.^10^

In this study, we applied our improved pipeline to the analysis of the human oral microbiota. The oral cavity is a large reservoir of bacteria of >700 species or phylotypes, and is profoundly relevant to host health and disease.^11–14^ Current studies reported that various oral symptoms such as aphthous stomatitis, oral ulcer, dry mouth, and pyostomatitis vegetans are frequently observed in inflammatory bowel disease (IBD) patients.^15–20^ IBD, including Crohn's disease (CD) and ulcerative colitis (UC), is a chronic, idiopathic, relapsing inflammatory disorder of the gastrointestinal tract.^21,22^ The most widely accepted mechanism of IBD pathogenesis includes inflammation due to altered host immune response in association with continuous stimulation from the resident gut microbiota.^23–28^ Many studies also revealed that the gut microbiota of IBD patients significantly differed from that of healthy controls (HCs), and is termed dysbiosis.^29–34^

Similarly, oral manifestations observed in IBD patients suggest the association of oral microbiota with such manifestations, yet-limited information exists about the oral microbiota of IBD patients. We characterized the salivary microbiota of IBD patients and HCs by barcoded pyrosequencing analysis of the bacterial 16S rRNA gene. We observed that the salivary microbiota in IBD patients significantly differed from that of HCs, and found particular bacterial species associated with dysbiosis. We also showed that the observed dysbiosis is strongly associated with elevated inflammatory response of several cytokines with depleted lysozyme in the saliva of IBD patients, some of which showed a strong correlation with the relative abundance of certain bacterial species. Thus, the present study demonstrates an association between dysbiosis of the salivary microbiota and change in the host's physiological state in IBD.

## 2. Material and methods

### 2.1. Patients and control groups

All participants of the CD, UC, and HC groups were informed of the purpose of this study, and written consent was obtained. This project was approved by the ethical committee of University of the Ryukyus. Metadata collected at the time of sampling included various demographics and a medication history for each patient (Supplementary Tables S1 and S2).

### 2.2. Sample collection and DNA extraction

Unstimulated saliva collected from subjects was immediately frozen by liquid nitrogen and stored in −80°C until use. Salivary genomic DNA was prepared according to the literature with minor modifications.^35^

Bacterial cells were harvested from 1 ml of saliva by centrifugation at 3300*g* for 10 min at 4°C. Bacterial pellets were suspended in 10 mM Tris–HCl/10 mM EDTA buffer and incubated with 15 mg/ml lysozyme (Sigma-Aldrich Co. LLC) for 1 h at 37°C. Purified achromopeptidase (Wako Pure Chemical Industries, Ltd.) was added to a final concentration of 2000 units/ml and samples were further incubated for 30 min. Ten percentage of (wt/vol) sodium dodecyl sulphate (SDS) and proteinase K (Merck Japan) were added to the suspension to final concentrations of 1% and 1 mg/ml, respectively, and samples were further incubated at 55°C for 1 h. The lysate was treated with phenol/chloroform/isoamyl alcohol (Life Technologies Japan, Ltd.) and centrifuged at 3300*g* for 10 min. DNA was precipitated by adding 1/10 volume of 3 M sodium acetate (pH 4.5) and 2 volumes of ethanol (Wako Pure Chemical Industries, Ltd.) to the supernatant. DNA was pelleted by centrifugation at 3300*g* for 15 min at 4°C. DNA pellets were rinsed with 75% ethanol, dried and dissolved in 10 mM Tris–HCl/1 mM EDTA (TE) buffer. DNA was further treated with 1 mg/ml RNase A (Wako Pure Chemical Industries, Ltd.) at 37°C for 30 min, and precipitated by adding equal volumes of 20% PEG solution (PEG6000-2.5M NaCl). DNA was pelleted by centrifugation at 8060*g* at 4°C, rinsed twice with 75% ethanol, dried, and dissolved in TE buffer.

### 2.3. Bacterial 16S rRNA gene-based analysis

#### 2.3.1. PCR amplification of the 16S rRNA gene V1–V2 region and barcoded 454 pyrosequencing

The hypervariable V1–V2 region of the 16S rRNA gene was amplified by PCR with barcoded 27Fmod and 338R primers.^10^ PCR was performed in 50 μl of 1× Ex Taq PCR buffer composed of 10 mM Tris–HCl (pH 8.3), 50 mM KCl, and 1.5 mM MgCl_2_ in the presence of 250 μM dNTP, 1 U Ex Taq polymerase (Takara Bio, Inc.), forward and reverse primers (0.2 μM) and ∼20 ng template DNA. Thermal cycling consisted of initial denaturation at 96°C for 2 min, followed by 25 cycles of denaturation at 96°C for 30 s, annealing at 55°C for 45 s and extension at 72°C for 1 min, and final extension at 72°C on a 9700 PCR system (Life Technologies Japan, Ltd.). Negative controls were treated similarly, except that no template DNA was added to the PCR reactions. PCR products of ∼370 bp were visualized by electrophoresis on 2% agarose gels, while negative controls failed to produce visible PCR products and were excluded from further analysis. PCR amplicons were purified by AMPure XP magnetic purification beads (Beckman Coulter, Inc.), and quantified using the Quant-iT PicoGreen dsDNA Assay Kit (Life Technologies Japan, Ltd.). Equal amounts of each PCR amplicon were mixed and then sequenced using either 454 GS FLX Titanium or 454 GS JUNIOR (Roche Applied Science).

#### 2.3.2 Analysis pipeline for 16S data

We developed and used an analysis pipeline for pyrosequencing data of the 16S rRNA gene V1–V2 region generated from oral microbiota. Based on sample specific barcodes, reads were assigned to each sample followed by the removal of reads lacking both forward and reverse primer sequences. Data were further denoised by removal of reads with average quality values <25 and possible chimeric sequences. For chimera checking and taxonomy assignment of the 16S rRNA data, we constructed our own databases from three publically available databases: Ribosomal Database Project (RDP) v. 10.27, CORE (http://microbiome.osu.edu/), and a reference genome sequence database obtained from the NCBI FTP site (ftp://ftp.ncbi.nih.gov/genbank/, December 2011). Reads having BLAST match lengths <90% with the representative sequence in the three databases were considered as chimeras and removed. Finally, filter-passed reads were used for further analysis after trimming off both primer sequences.

All of the 16S rRNA sequence data used in this study were deposited in DDBJ/GenBank/EMBL under accession numbers: DRA000984–DRA000986.

#### 2.3.3. Operational taxonomic unit clustering and UniFrac analysis

From the filter-passed reads, 3000 high-quality reads/sample were randomly chosen. The total reads (59 × 3000 reads) were then sorted according to average quality value and grouped into operational taxonomic units (OTUs) using UCLUST (http://www.drive5.com/) with a sequence identity threshold of 96%. Taxonomic assignments were made according to the best BLAST-hit phylotype. Weighted and unweighted UniFrac metrics^36^ were used to assess the diversity of the salivary microbiota between the CD, UC, and HC groups. UniFrac distances were based on the fraction of branch length shared between two communities within a phylogenetic tree constructed from the 16S rRNA gene sequences from all communities being compared.

### 2.4. Immunoassays

The centrifugal supernatant of unstimulated saliva was analyzed by the Luminex fluorescence technique, using the Bio-Plex Pro Human cytokine 27-Plex Assay (Bio-Rad Laboratories, Inc.) according to the manufacturer's instructions. LL-37 (cathelicidin, hCAP-18) levels were measured by ELISA using the Human LL-37 ELISA Kit (Hycult Biotech, Uden, The Netherlands). IgA levels were measured using the EIA-sIgA Test (MBL, Nagoya, Japan). Salivary lysozyme levels were measured using turbidimetric technique (SRL Inc., Japan). Total protein concentrations were measured by the Bradford protein assay using bovine serum albumin as the standard. In this study, saliva samples of only 15 HC, 14 CD, and 10 UC subjects were used for the assay of biomarkers, because the saliva from the other subjects was insufficient for measurement of all the indicated biomarkers.

### 2.5. Statistical analysis

All statistical analyses were conducted with R version 2.15.2. Microbial richness, evenness, and diversity were assessed using the R Vegan package. Depending on the normality of the data, the Student's *t*-test or Mann-Whitney's U-test was used to perform statistical analysis. *P*-values were corrected for multiple testing using the Benjamini–Hochberg method. Correlations between relative abundance of genera and immunological markers in saliva were calculated by Pearson correlation coefficients.

## 3. Results

### 3.1. Collection of 16S data

We surveyed the salivary microbiota of 21 CD patients, 14 UC patients, and 24 HCs, all of whom (including their relatives) are residents, lasting at least three generations, of the Okinawa area in Japan. The general and clinical parameters of the study populations are given in Supplementary Table S1, and individual details are shown in Supplementary Table S2. Sample-assigned pyrosequencing reads having both forward and reverse primer sequences accounted for ∼60% of the total number of reads. The 16S reads having average quality values <25 and possibly chimeric sequences represented 0.75 and 0.46% of the selected dataset, respectively. Finally, 506 133 high-quality 16S reads were obtained from 59 salivary samples. Sorting of the 16S reads by average quality value prior to clustering enabled selection of the representative sequence with the highest quality value among the 16S reads grouped in each OTU. On the other hand, the primer check step for removing reads lacking both primer sequences^10^ had the possibility to incorrectly remove reads containing V1–V2 regions longer than the maximum length of 431 bp in the filter-passed reads. This is because there are a few species with a V1–V2 region >431 bp (e.g. *Campylobacter rectus* has a length of 493 bp). Our primer check step did not significantly affect the present results because only one of the 177 000 raw reads examined hit to Campylobacter. However, to avoid the incorrect filtration of reads, we modified the primer check step so as not to remove reads having a length of >400 bp, even though they may not have both primer sequences.

### 3.2. Overall composition of the salivary bacterial communities

We evaluated the ecological features of the salivary bacterial communities of the CD, UC, and HC groups by a variety of indices at the OTU level.^37,38^ The results are summarized in Table 1. Species richness is the observed number of bacterial species assigned by OTUs detected in the samples. Richness estimates were obtained from the observed number of species by the extrapolation method using estimators such as the Chao1 and ACE indices. Evenness is the degree of homogeneity of abundance of the species detected in the samples. Diversity estimates were obtained from species richness and evenness by using several different indices, which exhibit different sensitivities to given factors, to confirm our results. The results suggested that there were no significant differences in the overall configuration of the salivary microbiota among the three groups (Table 1).

**Table 1.** OTU-based microbial richness and diversity across the HC, CD and UC groups

	HC	CD	UC
Diversity estimates			
Shannon Index	3.4 ± 0.1	3.4 ± 0.1	3.4 ± 0.1
Simpson Index	0.93 ± 0.01	0.93 ± 0.01	0.94 ± 0.01
Invsimpson Index	16.7 ± 1.1	16.7 ± 1.1	17.1 ± 1.4
Fisher alpha Index	26.8 ± 1.4	26.3 ± 1.4	24.8 ± 1.8
Evenness estimate			
Pielou's Index	0.7 ± 0.01	0.7 ± 0.01	0.71 ± 0.01
Richness estimates			
Number of OTUs	126 ± 5	124 ± 5	118 ± 7
chao1 Index	183 ± 8	183 ± 9	164 ± 13
ACE Index	182 ± 8	177 ± 8	165 ± 11

**Figure 1.**
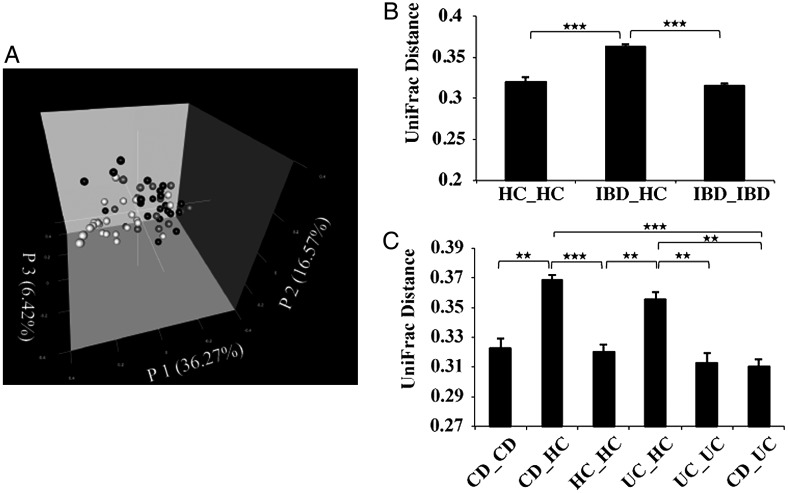
Analysis of the salivary microbiota of the HC, CD, and UC groups based on 16S data. (A) PCoA plot generated using weighted UniFrac metric. The three components explained 59.26% of the variance. White, grey, and black dots indicate HCs, UC, and CD samples, respectively. (B) Weighted UniFrac distance metric (a measure of differences in bacterial community structure) between HCs and the IBD (CD and UC) groups. (C) Weighted UniFrac distance metric between the HC, CD, and UC groups. Student's *t*-test was used; *P < 0.01, **P < 10^−5^, and ***P < 10^−10^; mean ± s.e.m.

We then compared the overall bacterial community composition using the UniFrac distance metric, a phylogenetic tree-based metric ranging from 0 (distance between identical communities) to 1 (distance between totally different communities). A principal coordinate analysis (PCoA) plot based on the weighted UniFrac metric revealed clear clustering of most IBD samples apart from the HC samples, indicating the difference in microbial communities between the two groups (Fig. 1A). A bar chart more clearly shows the significant difference in microbiota composition between the IBD and HC groups (Fig. 1B). Comparison of the salivary microbiota of HCs with that of the CD and UC groups indicated that the microbiota of HCs significantly differs from both of them, and no significant difference was found between the UC and CD groups (Fig. 1C). Similar results were obtained using the unweighted UniFrac metric with lower statistical significance than that of the weighted UniFrac metric (Supplementary Fig. S1). These data suggest that species abundance, rather than species diversity, largely contributes to the observed differences in salivary microbiota between the HC and IBD groups.

Although the average age was considerably different between HCs and the IBD patients, weighted UniFrac distance analysis of 10 selected healthy subjects (average age 25.0 yr), 10 IBD patients (average age 28.7 yr, which matched the selected HC group), and the remaining 25 IBD patients (average age 54.6 yr) showed results similar to that of the total samples (Supplementary Fig. S2). Moreover, there was no significant difference between the two IBD subgroups. These data suggest that age might not affect the observed dysbiosis of the salivary microbiota of the IBD patients.

### 3.3. Differences in salivary microbiota composition between the HC, CD, and UC groups

**Figure 2.**
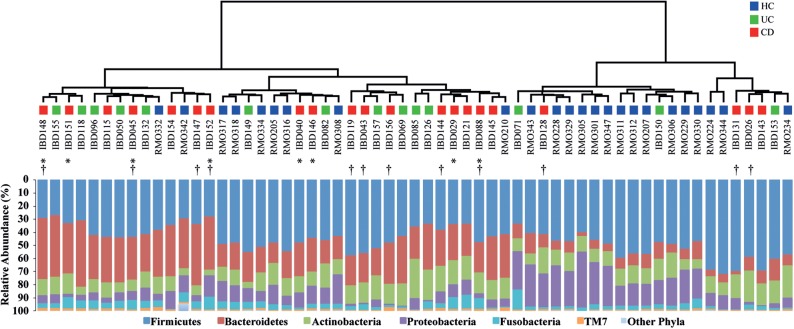
Cluster dendrogram generated using weighted UniFrac metric. Bar charts show the relative abundance of different phyla across the CD, UC and HC samples. Asterisks indicate samples taken during the active phase of CD. Dagger indicates anti-TNF-α antibody treated CD.

The final dataset of the examined CD, UC, and HC groups (*n* = 59) consisted of 177 000 reads and included representatives of 12 bacterial phyla (Fig. 2; Supplementary Fig. S3 and Table S3). The majority of the 16S reads were classified into only five phyla: Firmicutes (46.5%), Bacteroidetes (22.3%), Actinobacteria (13.7%), Proteobacteria (12.5%), and Fusobacteria (4.2%). TM7, SR1, Spirochaetes, Synergistetes, Tenericutes, and Cyanobacteria were also detected and collectively represented <1% of the total reads analyzed. Analysis at the phylum level showed that the relative abundance of Bacteroidetes was significantly higher in both the CD and UC groups as compared with HCs (*P* < 0.01), while that of Proteobacteria was significantly lower in both the CD and UC groups as compared with HCs (*P* < 0.01). No significant difference at the phylum level was observed between the UC and CD groups, which was consistent with the results of the UniFrac distance analysis.

**Figure 3.**
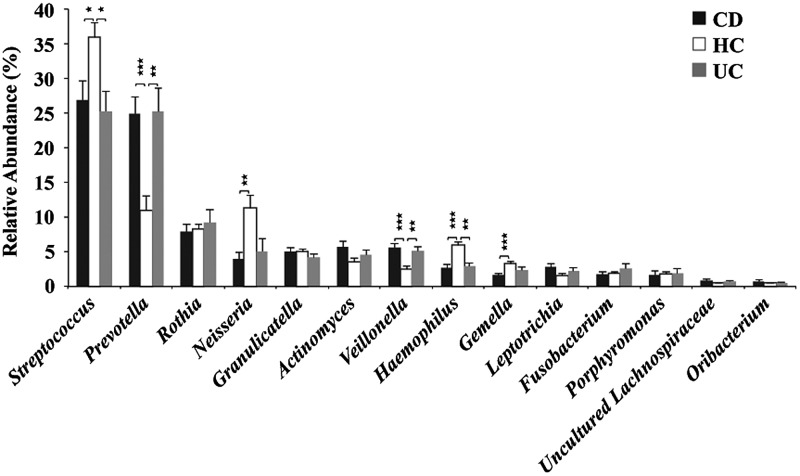
Mean genus abundance in the CD, UC and HC groups. Plotted values are the mean abundance of the 14 most abundant genera in each group. Welch's test with BH adjustment was used; *P < 0.05, **P < 0.01, and ***P < 0.001; mean ± s.e.m.

In total, 107 bacterial genera were identified (at 95% identity), accounting for 97.8% of the total dataset. The remaining unclassified sequences (2.2%) were assigned to higher level taxa. Fourteen genera, including *Streptococcus*, *Prevotella*, *Rothia*, *Neisseria*, *Granulicatella*, *Actinomyces*, *Haemophilus*, *Veillonella*, *Gemella*, *Leptotrichia*, *Fusobacterium*, *Porphyromonas*, *Uncultured Lachnospiraceae*, and *Oribacterium*, predominated accounting for 92.7% of the total dataset. Other genera represented <0.5% each (Fig. 3; Supplementary Table S3). Two genera, *Prevotella* (phy. Bact.) and *Veillonella* (phy. Firm.), were significantly higher in both the CD and UC groups compared with HCs (*P* < 0.01). Two genera, *Streptococcus* (phy. Firm.) and *Haemophilus* (phy. Prot.), were significantly lower in both the CD and UC groups as compared with HCs (*P* < 0.05 and 0.01, respectively). Two other genera, *Neisseria* (phy. Prot.) and *Gemella* (phy. Firm.), were also found to be significantly lower only in the CD group as compared with HCs (*P* < 0.01 and 0.001, respectively). These results indicate that the relative increase of Bacteroidetes in IBD patients was mainly due to the increase of *Prevotella*, and the relative decrease of Proteobacteria in IBD patients was mainly due to the decrease of *Neisseria* and *Haemophilus*. No significant difference in the relative abundance of either Gram-positive or Gram-negative bacteria was observed among the three groups (Supplementary Table S3).

Clustering of all reads using a 96% pairwise-identity cutoff generated 1257 OTUs, of which only 40 OTUs represented 67.2% of the total reads analyzed. The remaining OTUs were present at relative abundance levels <0.5% of the total dataset (Supplementary Table S4). The relative abundance of several OTUs belonging to the genera *Streptococcus*, *Prevotella*, *Veillonella*, *Nesisseria*, *Haemophilus*, and *Gemella* showed significant differences in IBD patients as compared with HCs. These results were concordant with those detected at the genus level. Among the abundant OTUs, those most closely assigned to *Prevotella melaninogenica*, *Veillonella* sp. oral taxon 158, *Streptococcus mitis*, *Gemella sanguinis*, *Neisseria mucosa*, and *Haemophilus parainfluenzae* showed significant differences in relative abundance between the HC and IBD groups (Supplementary Table S4).

### 3.4. Salivary immunological biomarkers in the HC, CD, and UC groups

We evaluated the inflammatory state, considering its influence on shaping the salivary microbiota, in saliva of the CD and UC patients as compared with that of HCs. The analysis was performed by measuring secretory IgA, cytokines, and enzymes including lysozyme in unstimulated saliva of 15 HC, 14 CD, and 10 UC individuals (Supplementary Table S5 and Fig. S4). There was no significant difference in the total protein concentration in saliva of the CD and UC patients as compared with that of HCs (*P* = 0.112 and 0.192, respectively). The lysozyme level was significantly lower in saliva of both the CD and UC groups as compared with HCs (*P* < 0.01). On the other hand, the levels of IgA and LL37 in both CD and UC groups were higher than that of HCs with statistical significance. The use of Luminex technology was highly sensitive in measuring cytokines from small volumes of saliva samples. In saliva of the CD and UC groups, the level of IL-1β was significantly higher as compared with HCs (*P* < 0.05 and <0.01, respectively). The levels of IL-6, IL-8, and MCP-1 were significantly higher only in saliva of the UC group, while elevated TNF-α level was found only in the CD group with statistical significance. The levels of IgA and MCP-1 in the UC group were significantly higher than those in the CD group. These data indicate that the oral cavity of IBD patients is usually in the inflammatory state, and the levels tend to be slightly higher in the UC group than the CD group.

### 3.5. Composition of the salivary microbiota in relation to immunological biomarkers

**Figure 4.**
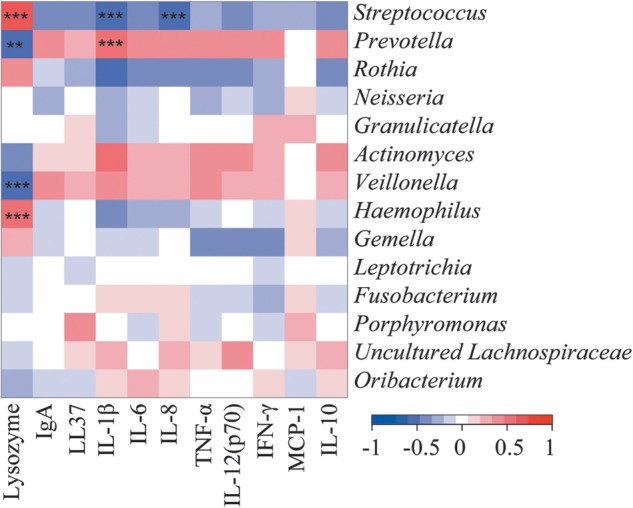
Correlation between the relative abundance of predominant genera and the level of immunological biomarkers in the saliva of IBD patients. Pearson product moment correlation coefficients are represented by colour ranging from blue, negative correlation (−1), to red, positive correlation (1). Normalized values of immunological biomarkers by total protein amount were used in this analysis. Significant correlations after *P*-value adjustment are marked by *P < 0.05, **P < 0.01, and ***P < 0.001.

We searched for correlations between the relative abundance of dominant bacterial genera and the measured biomarkers in the saliva of 39 subjects (Supplementary Table S5). The results are shown in Fig. 4. The relative abundance of *Streptococcus* negatively correlates with IL-1β and IL-8 (*r* = −0.54 and −0.51, respectively, *P* < 0.001), while it positively correlates with lysozyme (*r* = 0.63, *P* < 0.001). On the other hand, the abundance of *Prevotella* positively correlates with IL-1β (*r* = 0.58, *P* < 0.001) but negatively correlates with lysozyme (*r* = −0.54, *P* < 0.01). The relative abundance of *Veillonella* negatively correlates with lysozyme (*r* = −0.54, *P* < 0.001), while *Haemophilus* positively correlates with lysozyme (*r* = 0.58, *P* < 0.001). Linear regressions also validated correlations between the relative abundance of *Streptococcus* and *Prevotella* and the levels of lysozyme and IL-1β, and between the relative abundance of *Veillonella* and *Haemophilus* and the level of lysozyme (Supplementary Fig. S5). On the whole, *Prevotella*, *Actinomyces*, *Veillonella*, and *Lachnospiracea* tended to positively correlate, while *Streptococcus*, *Rothia*, *Neisseria*, *Haemophilus*, and *Gemella* tended to negatively correlate with elevated cytokines in saliva of IBD patients.

### 3.6. Validation of 16S pyrosequencing data by targeted quantitative PCR

**Figure 5.**
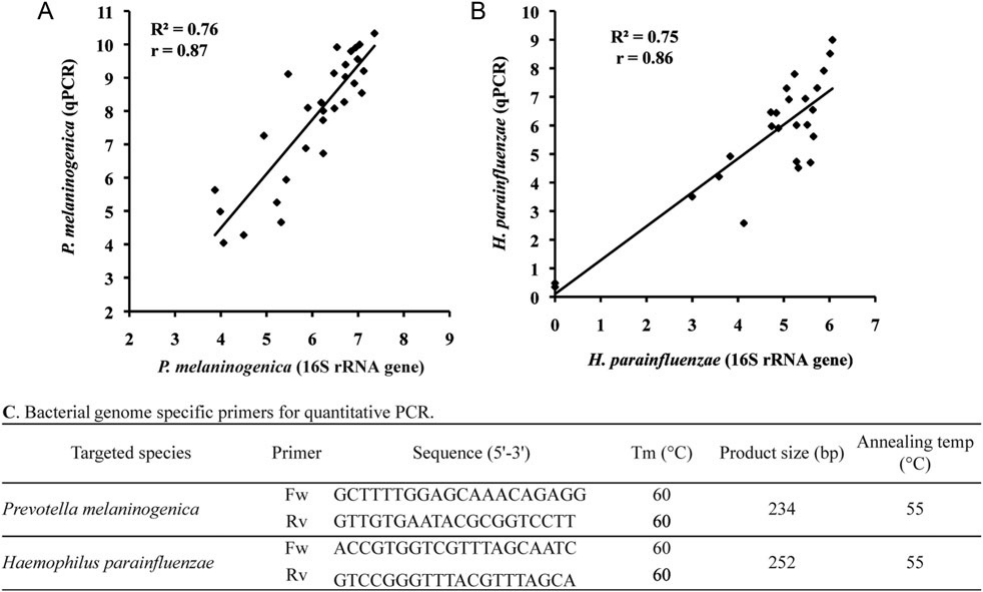
Correlation between the 16S rRNA pyrosequencing and qPCR data. The results are shown in (A) for *P. melaninogenica* and (B) for *H. parainfleuenzae*. The *y*-axis represents the copy number per nanogram of bacterial DNA obtained from qPCR data, transformed by the inverse hyperbolic sine method. The *x*-axis represents the number of reads assigned as bacterial spp. obtained from the pyrosequencing data, transformed by inverse hyperbolic sine method. Pearson product moment correlation coefficient (*r*) on transformed data (using inverse hyperbolic sine transformation) is shown. (C) Primer sequences and PCR conditions used for qPCR experiments are shown.

We designed specific PCR primers for quantitative PCR (qPCR) targeting genomes of *P. melaninogenica* and *H. parainfluenzae*, which showed significant differences between HCs and IBD patients by 16S pyrosequencing analysis (Supplementary Table S4). Using these primers, we found strong correlations between 16S-based and qPCR data for the quantification of *P. melaninogenica* (*r* = 0.87, *P* < 0.001) and *H. parainfluenzae* (*r* = 0.86, *P* < 0.001), indicating the quantitative accuracy of our 16S pyrosequencing-based results (Fig. 5).

## 4. Discussion

### 4.1. Bacterial 16S rRNA-based pyrosequencing analysis

In this study, we used targeted amplicon sequencing of the 16S rRNA gene hypervariable V1–V2 region to evaluate bacterial composition at finer taxonomic levels. The use of primer 27Fmod enabled us to reduce underestimation of the relative abundance of *Bifidobacterium* species that predominate human microbiota, and thus the quantitative accuracy of the overall bacterial composition was greatly improved.^10,39^ One limitation of clustering the 16S reads using the UCLUST program is selection of the representative sequence for each OTU. The quality of the representative sequence is not always the highest in the OTU, which affects the BLAST identity, *E*-value and score, sometimes providing inappropriate results for taxonomic assignment of the OTUs. We overcame this limitation by sorting the 16S reads by their average quality values prior to clustering, leading to 16S reads with the highest quality being selected as the representative sequence for each OTU. Our 16S-based results were also validated by strongly correlating with the qPCR data targeting bacterial species showing significant changes between HC and IBD samples (Fig. 5). In addition, clustering of the reads was performed with a 96% pairwise-identity cutoff to reduce overestimation of the number of bacterial species (or OTUs) largely due to 454 pyrosequencing errors.^10,40^ Clustering with a 96% pairwise-identity cutoff should be applied for pyrosequencing reads obtained from other types of human microbiota.

### 4.2. Salivary microbiota composition in IBD patients

The abundant bacterial groups in the salivary microbiota detected in this study were similar to those previously reported,^41–44^ but the compositions differed from those observed in plaque microbiota.^44^ Our data clearly showed a significant difference in salivary microbiota composition between HCs and IBD patients. Shifts in oral microbiota composition were also observed in several oral manifestations such as dental caries,^45^ periodontitis,^46^ and oral squamous cell carcinoma.^47^ Moreover, various components of the oral microbiota have been implicated in systemic diseases such as pancreatic disease including pancreatic cancer,^48^ atherosclerosis,^49^ bacteremia,^50^ and endocarditis.^51^

Altered bacterial community structure in the gut microbiota of IBD patients is a common finding in comparison with that of healthy subjects. Previous studies showed overall structural changes as well as reduced species richness of the gut microbiota in IBD patients.^29–33^ It is likely that the high microbial richness and diversity characterizing healthy microbiota may have a protective effect on humans. Unlike the gut microbiota of IBD patients, our estimates using several metrics revealed that microbial richness and diversity in the salivary microbiota of IBD patients was similar to that of HCs, despite significant changes in community structure (Fig. 1). These data suggest that the extent of the changes in the salivary microbiota is less than that in the gut microbiota of IBD patients.

Our data indicated a significant increase of the genus *Prevotella* in the salivary microbiota of IBD patients, in which its relative abundance was almost equivalent to that of reduced *Streptococcus*, which is most abundant in healthy salivary microbiota (Fig. 3). *Prevotella* is a Gram-negative, obligate anaerobe, and a member of the prevalent genera in the human microbiome.^52^ Some *Prevotella* species were similarly increased, distinguishable from opportunistic infections, in bacterial vaginosis,^53^ esophagitis,^54^ antral gastritis,^55^ and saliva of caries-active subjects.^45^ These data suggest that the increase of *Prevotella,* with concurrently decreased *Streptococcus*, is clearly related with abnormal physiologies in IBD patients. The relative abundance of total Gram-positive and Gram-negative bacteria showed no significant difference between HCs and IBD patients (Supplementary Table S3). From these results, Gram-stain properties of bacterial surface structures may not be related with dysbiosis of IBD salivary microbiota, unlike the association of Gram-negative oral bacteria with dysbiosis observed in subgingival microbiota in periodontitis.^56^

### 4.3. Salivary microbiota associated with immunological biomarkers

Saliva contains a variety of components such as cytokines, immunoglobulins, and antimicrobial proteins involved in host defence mechanisms for maintaining oral and systemic health.^57^ Alteration of the salivary microbiota in IBD patients suggests the occurrence of inflammatory immune responses in the oral cavity of IBD patients as intestinal inflammation associated with aberrant gut microbiota of IBD.^23–26^ Our data showed that the levels of many salivary cytokines and IgA were significantly higher in both CD and UC patients than those observed in HCs, indicating that inflammatory responses are elicited in the oral cavity of the patients. Similarly, elevated salivary IL-1β, IL-6, and TNF-α levels in CD patients and an elevated IL-8 level in the saliva of patients with bowel disease were also reported.^58,59^ Unexpectedly, the elevated level of inflammatory biomarkers in UC patients was similar to or slightly higher than that observed in CD patients, regardless of differences in disease states between IBD patients (Supplementary Fig. S4 and Table S5). Salivary IgA induction was observed in CD patients with oral symptoms but not in those without oral symptoms.^60^ The elevated level of IgA in most IBD patients’ saliva examined suggests that those patients may have oral manifestations, however, we did not have access to their oral health clinical records.

Salivary lysozyme levels were significantly reduced in both CD and UC patients as compared with that of HCs. Lysozyme is an antimicrobial protein, expressed by various cells including neutrophils, macrophages, and epithelial cells. It is abundant in saliva and plays an important role in the host constitutive defence system.^61^ It has been reported that salivary lysozyme was significantly lower in patients with gingivitis and periodontitis as compared with healthy subjects.^62^ In contrast, faecal lysozyme levels were significantly elevated in IBD patients.^63^ Further analysis will be required to elucidate the difference in lysozyme levels between saliva and the intestine.

Lysozyme exclusively catalyses hydrolysis of Gram-positive bacterial cell wall. However, lysozyme can also be bactericidal for Gram-negative bacteria *in vivo* through synergistic action with salivary lactoferrin in the normal state.^64^ Therefore, this *in vitro* specificity of lysozyme activity may not be largely involved in the dysbiosis of salivary microbiota in IBD patients, in which the abundance of Gram-positive bacteria was not significantly different as compared with HCs (Supplementary Table S3).

There were several subgroups of patients dependent on different medical treatments, and patients with different states of disease (Supplementary Tables S1 and S2). In addition, Infliximab (anti-TNF-α antibody) therapy is commonly used for IBD patients, but up to one-third of the patients have been shown not to respond.^65^ Therefore, it was very difficult to precisely evaluate the differences in microbiota structure and biomarker levels between the subgroups. Nevertheless, phylogenetic analysis based on the weighted UniFrac distance metric did not show discrete clustering of particular subgroups, such as CD patients with or without Infliximab treatment and active CD, or CD in remission, suggesting limited contributions from the patients' disease state or medical treatment to the overall microbiota structure (Fig. 2).

Strong correlations between some inflammatory biomarkers and salivary microbiota compositions were revealed (Fig. 4). The lower lysozyme and elevated IL-1β, IL-8, IgA and several other biomarkers were likely to be synergistically or interactively associated with the abundance of the four dominant genera, *Streptococcus*, *Prevotella*, *Veillonella*, and *Haemophilus*. Interactions between these microbes and other species may also be involved in the dysbiosis of salivary microbiota of IBD patients.

Finally, it is still unknown whether the inflammatory state in the oral cavity of IBD patients is the cause or a consequence of imbalances in the salivary microbiota, and which local (the oral cavity) or systemic (the gut) immune response is more responsible for the observed dysbiosis of salivary microbiota. Our results strongly suggest the existence of certain defined mechanisms by which aberrant, but similar, salivary microbiota among IBD patients is formed. The human gut microbiota is gradually shaped to its matured assemblage in a few years after birth, with temporal changes in the diversity and rank of dominant species largely dependent on diet and host physiological state.^66^ Salivary microbiota may also be established similar to gut microbiota. Since >1000 ml of saliva is produced per day in the average adult and it always flows into the gastrointestinal tract, bacteria in saliva also have many opportunities to reach the intestine. Therefore, it can be postulated that salivary microbiota affects the development of gut microbiota to some extent. To evaluate this hypothesis, it is necessary to investigate the progression of infant salivary microbiota and the oral inflammatory state. Additionally, further studies such as comparison of the salivary microbiota between IBD and other diseases will provide informative sources for discovering non-invasive salivary biomarkers specific to IBD.

## Supplementary data

Supplementary data are available at www.dnaresearch.oxfordjournals.org.

## Funding

This work was supported in part by the global COE project of ‘Genome Information Big Bang’ from the Ministry of Education, Culture, Sports, Science, and Technology (MEXT) of Japan to M.H. and K.O., a research project grant from Azabu University to H.M., a grant from the Core Research for Evolutional Science and Technology (CREST) program of the Japan Science and Technology Agency (JST) to K.O., and Scientific Research B (No. 22370087) to H.I. and (Nos. 21370108 and 24370099) to H.O. from the Japan Society for the Promotion of Science (JSPS). H.S.S. acknowledges the fellowship from MEXT.

## Supplementary Material

Supplementary Data
