# Supplementary material for: Dysbiosis of Salivary Microbiota in Inflammatory Bowel Disease and Its Association With Oral Immunological Biomarkers
Source: DNA Res. 2013 Sep 7;21(1):15–25. doi: 10.1093/dnares/dst037 (PMC3925391; doi:10.1093/dnares/dst037)
Supplement: Supplementary Data [file supp_21_1_15__index.html]

Dysbiosis of Salivary Microbiota in Inflammatory Bowel Disease and Its Association With Oral Immunological Biomarkers — Dysbiosis of Salivary Microbiota in Inflammatory Bowel Disease and Its Association With Oral Immunological Biomarkers — Supplementary Data 

# Dysbiosis of Salivary Microbiota in Inflammatory Bowel Disease and Its Association With Oral Immunological Biomarkers

## Supplementary Data

Supplementary Data

**Files in this Data Supplement:**

- Supplementary Data - Doc file
- Supplementary Figure 1 - tif file
- Supplementary Figure 2 - tif file
- Supplementary Figure 3 - tif file
- Supplementary Figure 4 - tif file
- Supplementary Figure 5 - tif file
