# Supplementary material for: Dysbiosis of Salivary Microbiota in Inflammatory Bowel Disease and Its Association With Oral Immunological Biomarkers
Source: DNA Res. 2013 Sep 7;21(1):15–25. doi: 10.1093/dnares/dst037 (PMC3925391; doi:10.1093/dnares/dst037)
Supplement: Supplementary Data [file supp_dst037_dst037supp.doc]

**Supplementary tables**

Table S1. Demographic and clinical characteristics of the study groups

|  | HC (n=24) | CD (n=21) | UC (n=14) |
| --- | --- | --- | --- |
| - Gender |  |  |  |
| Males, n (%) | 15 (62.5) | 18 (85.7) | 5 (35.7) |
| - Age, years | 22±0.8 | 40.1±2.7 | 57.9±5.1 |
| - Active disease, n (%) | - | 8 (38.1) | 3 (21.4) |
| - IBD treatment stategies, n (%) |  |  |  |
| Anti-inflammatory drugs | - | 17 (81) | 12 (85.7) |
| Immunosuppresives | - | 4 (19) | 0 (0) |
| Anti TNF-α | - | 12 (57.1) | 0 (0) |
| - Crohn's disease types, n (%) |  |  |  |
| Ileal | - | 1 (4.8) | - |
| Ileocolonic | - | 19 (90.5) | - |
| Colonic | - | 1 (4.8) | - |
| - Ulcerative colitis types, n (%) |  |  |  |
| Proctitis | - | - | 4 (28.6) |
| Left-sided colitis | - | - | 6 (42.9) |
| Pancolitis | - | - | 4 (28.6) |

Table S2. Patient and healthy control information

| Sample ID | Condition | Gender | Age (years) | Type | IBD treatment strategies | | | | | | CD=IOIBD  /UC=UC-DAI |
| --- | --- | --- | --- | --- | --- | --- | --- | --- | --- | --- | --- |
|  |  |  |  |  | Anti-inflammatory drugs | | | | Immunosuppressives | Anti TNF-α |  |
|  |  |  |  |  | Oral PSL | | PSL Enema | Oral 5-ASA | AZ | IFX |  |
| IBD026 | CD | M | 52 | Ileocolic | - | | - | ○ | - | ○ | IOIBD 0 (Remission) |
| IBD029 | CD | M | 50 | Ileocolic | - | | - | ○ | - | - | IOIBD 3 (Active) |
| IBD040 | CD | M | 38 | Ileocolic | - | | - | - | - | - | IOIBD 2 (Active) |
| IBD043 | CD | M | 57 | Ileocolic | - | | - | ○ | ○ | ○ | IOIBD 1 (Remission) |
| IBD045 | CD | M | 39 | Ileocolic | - | | - | ○ | - | ○ | IOIBD 3 (Active) |
| IBD088 | CD | M | 40 | Ileocolic | - | | - | ○ | ○ | ○ | IOIBD 6 (Active) |
| IBD115 | CD | M | 48 | Ileocolic | - | | - | ○ | ○ | - | IOIBD 1 (Remission) |
| IBD119 | CD | F | 36 | Ileocolic | - | | - | ○ | - | ○ | IOIBD 0 (Remission) |
| IBD121 | CD | M | 52 | Ileocolic | - | | - | ○ | - | - | IOIBD 1 (Remission) |
| IBD128 | CD | M | 68 | Ileocolic | - | | - | ○ | - | ○ | IOIBD 0 (Remission) |
| IBD131 | CD | M | 19 | Ileocolic | - | | - | ○ | ○ | ○ | IOIBD 1 (Remission) |
| IBD143 | CD | M | 42 | Ileocolic | - | | - | - | - | - | IOIBD 1 (Remission) |
| IBD144 | CD | M | 32 | Ileocolic | - | | - | - | - | ○ | IOIBD 1 (Remission) |
| IBD145 | CD | F | 26 | Ileocolic | ○ | | - | - | - | - | IOIBD 0 (Remission) |
| IBD146 | CD | F | 31 | Ileum | - | | - | ○ | - | - | IOIBD 2 (Active) |
| IBD147 | CD | M | 17 | Ileocolic | - | | - | ○ | - | ○ | IOIBD 1 (Remission) |
| IBD148 | CD | M | 35 | Ileocolic | - | | - | ○ | - | ○ | IOIBD 2 (Active) |
| IBD151 | CD | M | 36 | Ileocolic | - | | - | ○ | - | - | IOIBD 2 (Active) |
| IBD152 | CD | M | 41 | Colon | - | | - | ○ | - | ○ | IOIBD 2 (Active) |
| IBD154 | CD | M | 36 | Ileocolic | - | | - | ○ | - | - | IOIBD 1 (Remission) |
| IBD156 | CD | M | 48 | Ileocolic | - | | - | ○ | - | ○ | IOIBD 1 (Remission) |
| IBD050 | UC | F | 34 | Left-sided colitis | - | | ○ | ○ | - | - | UC-DAI Mild |
| IBD069 | UC | F | 71 | Proctitis | ○ | | - | ○ | - | - | UC-DAI Mild |
| IBD071 | UC | M | 78 | Colectomy | - | | - | - | - | - | UC-DAI Mild |
| IBD082 | UC | F | 56 | Proctitis | - | | BMS（Supp） | ○ | - | - | UC-DAI Mild |
| IBD085 | UC | F | 41 | Pancolitis | - | | - | ○ | - | - | UC-DAI Mild |
| IBD096 | UC | F | 23 | Proctitis | - | | ○ | ○ | - | - | UC-DAI Mild |
| IBD118 | UC | M | 65 | Left-sided colitis | - | | ○ | ○ | - | - | UC-DAI Moderate |
| IBD126 | UC | F | 79 | Proctitis | - | | - | ○ | - | - | UC-DAI Mild |
| IBD132 | UC | F | 55 | Left-sided colitis | - | | ○ | ○ | - | - | UC-DAI Mild |
| IBD149 | UC | M | 47 | Pancolitis | - | | - | ○ | - | - | UC-DAI Mild |
| IBD150 | UC | F | 41 | Left-sided colitis | - | | ○ | ○ | - | - | UC-DAI Moderate |
| IBD153 | UC | F | 85 | Colectomy | - | | - | - | - | - | UC-DAI Mild |
| IBD155 | UC | M | 58 | Left-sided colitis | - | | - | ○ | - | - | UC-DAI Mild |
| IBD157 | UC | M | 78 | Left-sided colitis | - | | - | ○ | - | - | UC-DAI Moderate |
| RMO205 | HC | M | 21 |  |  | |  |  |  |  |  |
| RMO207 | HC | M | 31 |  |  | |  |  |  |  |  |
| RMO210 | HC | F | 23 |  |  | |  |  |  |  |  |
| RMO224 | HC | M | 29 |  |  | |  |  |  |  |  |
| RMO228 | HC | M | 25 |  |  | |  |  |  |  |  |
| RMO229 | HC | M | 21 |  |  | |  |  |  |  |  |
| RMO234 | HC | M | 28 |  |  | |  |  |  |  |  |
| RMO301 | HC | M | 20 |  |  | |  |  |  |  |  |
| RMO305 | HC | M | 20 |  |  | |  |  |  |  |  |
| RMO306 | HC | F | 20 |  |  | |  |  |  |  |  |
| RMO308 | HC | M | 20 |  |  | |  |  |  |  |  |
| RMO311 | HC | F | 20 |  |  | |  |  |  |  |  |
| RMO312 | HC | M | 19 |  |  | |  |  |  |  |  |
| RMO316 | HC | M | 20 |  |  | |  |  |  |  |  |
| RMO317 | HC | F | 20 |  |  | |  |  |  |  |  |
| RMO318 | HC | M | 20 |  |  | |  |  |  |  |  |
| RMO329 | HC | M | 20 |  |  | |  |  |  |  |  |
| RMO330 | HC | M | 20 |  |  | |  |  |  |  |  |
| RMO332 | HC | M | 20 |  |  | |  |  |  |  |  |
| RMO334 | HC | F | 32 |  |  | |  |  |  |  |  |
| RMO342 | HC | F | 19 |  |  | |  |  |  |  |  |
| RMO343 | HC | F | 19 |  |  | |  |  |  |  |  |
| RMO344 | HC | F | 20 |  |  | |  |  |  |  |  |
| RMO347 | HC | F | 20 |  |  | |  |  |  |  |  |
| PSL: Prednisolone, BMS: Betamethasone, 5-ASA: 5-Aminosalicylates, AZ: Azathioprine, IFX: Infliximab (anti TNF-α antibody). | | | | | | | | | | | |
| IOIBD: International Organization of Inflammatory Bowel Disease. | | | | | | UC-DAI: Ulcerative colitis Disease Activity Index | | | | | |

Table S3. Species abundance in the salivary microbiota of the HC, CD and UC groups at the phylum, genus, and Gram-staining levels

| Bacterial species | | Relative abundance | | | P-value | | |
| --- | --- | --- | --- | --- | --- | --- | --- |
|  |  | HC | CD | UC | CD_HC | UC_HC | CD_UC |
| Phyla | Firmicutes | 50±1.9 | 45±2.6 | 42.8±2.6 | 0.203 | 0.063 | 0.993 |
|  | Bacteroidetes | 14.3±2 | 27.5±2.6 | 28.1±3.2 | **0.0009** | **0.005** | 0.993 |
|  | Actinobacteria | 12.5±0.8 | 14.5±1 | 14.7±1.9 | 0.203 | 0.359 | 0.993 |
|  | Proteobacteria | 18.8±2.1 | 7.7±1.4 | 8.9±2 | **0.0004** | **0.005** | 0.993 |
|  | Fusobacteria | 3.5±0.4 | 4.6±0.7 | 4.8±1 | 0.230 | 0.320 | 0.993 |
|  | TM7 | 0.5±0.1 | 0.6±0.1 | 0.6±0.1 | 0.287 | 0.359 | 0.993 |
| Genera | Streptococcus (Firm) | 35.9±2.2 | 26.9±2.8 | 25.3±2.8 | **0.034** | **0.0197** | 0.933 |
|  | Prevotella (Bact) | 11±2 | 24.9±2.4 | 25.3±3.4 | **0.0005** | **0.007** | 0.933 |
|  | Rothia (Actino) | 8.3±0.7 | 7.9±1.1 | 9.2±1.9 | 0.882 | 0.704 | 0.933 |
|  | Neisseria (Prot) | 11.3±1.8 | 4±0.9 | 5.1±1.8 | **0.003** | 0.061 | 0.933 |
|  | Granulicatella (Firm) | 5±0.4 | 5.1±0.5 | 4.2±0.6 | 0.919 | 0.388 | 0.880 |
|  | Actinomyces (Actino) | 3.5±0.6 | 5.7±0.9 | 4.5±0.7 | 0.094 | 0.388 | 0.880 |
|  | Veillonella (Firm) | 2.5±0.4 | 5.6±0.5 | 5.1±0.6 | **0.0002** | **0.007** | 0.933 |
|  | Haemophilus (Prot) | 5.9±0.5 | 2.7±0.5 | 2.9±0.5 | **0.0005** | **0.004** | 0.933 |
|  | Gemella (Firm) | 3.3±0.3 | 1.6±0.2 | 2.4±0.4 | **0.0007** | 0.208 | 0.880 |
|  | Leptotrichia (Fuso) | 1.6±0.2 | 2.8±0.5 | 2.2±0.5 | 0.094 | 0.388 | 0.933 |
|  | Fusobacterium (Fuso) | 1.9±0.2 | 1.7±0.4 | 2.6±0.7 | 0.882 | 0.405 | 0.880 |
|  | Porphyromonas (Bact) | 1.8±0.3 | 1.7±0.6 | 1.8±0.7 | 0.919 | 0.957 | 0.933 |
|  | Uncultured Lachnospiraceae (Firm) | 0.5±0.1 | 0.8±0.2 | 0.7±0.1 | 0.232 | 0.111 | 0.933 |
|  | Oribacterium (Firm) | 0.4±0.1 | 0.8±0.2 | 0.5±0.1 | 0.125 | 0.402 | 0.880 |
| Gram-positive bacteria | | 63.1 | 60.2 | 58.1 | 0.459 | 0.196 | 0.639 |
| Gram-negative bacteria | | 36.5 | 39.7 | 41.8 | 0.407 | 0.166 | 0.644 |
| Relative abundance expressed as % (mean ± s.e.m.) | | | | | | | |
| P-value, Welch's test with BH adjustment for multiple comparisons (significant values in bold) | | | | | | | |

Table S4. Species abundance in the salivary microbiota of the HC, CD, and UC groups at the OTU level

| OTU number | Best BLAST-Hit Assignment | | | | Relative Abundance | | | P-value | | |
| --- | --- | --- | --- | --- | --- | --- | --- | --- | --- | --- |
|  | Phylum | Genus | Species | Identity in % | HC | CD | UC | CD_HC | UC_HC | CD_UC |
| OTU00144 | Actinobacteria | Actinomyces | Actinomyces graevenitzii | 99.34 | 31±8 | 29±10 | 23±9 | 0.858 | 0.722 | 0.967 |
| OTU00205 |  |  | Actinomyces sp. oral taxon 180 | 98.71 | 23±5 | 50±13 | 40±8 | 0.139 | 0.070 | 0.967 |
| OTU00003 |  | Rothia | Rothia mucilaginosa | 100 | 169±19 | 173±24 | 236±51 | 0.976 | 0.556 | 0.967 |
| OTU00143 |  |  | Rothia mucilaginosa | 95.53 | 47±14 | 33±10 | 11±9 | 0.712 | 0.063 | 0.967 |
| OTU00036 |  |  | Rothia sp. oral taxon 188 | 100 | 30±5 | 23±6 | 25±9 | 0.154 | 0.241 | 0.967 |
| OTU00122 | Bacteroidetes | Porphyromonas | Porphyromonas catoniae | 99.05 | 38±7 | 32±12 | 32±11 | 0.295 | 0.506 | 0.967 |
| OTU00194 |  | Prevotella | Prevotella DO039 | 99.05 | 10±3 | 34±12 | 35±13 | 0.070 | **0.047** | 0.967 |
| OTU00160 |  |  | Prevotella histicola | 100 | 33±10 | 93±27 | 136±54 | 0.073 | 0.070 | 0.967 |
| OTU00066 |  |  | Prevotella melaninogenica | 99.37 | 63±15 | 125±25 | 104±29 | 0.129 | 0.302 | 0.967 |
| OTU00262 |  |  | Prevotella melaninogenica | 99 | 23±10 | 72±21 | 58±18 | **0.028** | 0.298 | 0.967 |
| OTU00724 |  |  | Prevotella melaninogenica | 97.48 | 15±3 | 60±12 | 47±11 | **0.011** | **0.047** | 0.967 |
| OTU00449 |  |  | Prevotella melaninogenica | 95.58 | 30±10 | 20±12 | 23±12 | 0.105 | 0.748 | 0.967 |
| OTU00417 |  |  | Prevotella melaninogenica | 98.63 | 13±4 | 18±6 | 37±9 | 0.712 | 0.070 | 0.967 |
| OTU00193 |  |  | Prevotella pallens | 99.68 | 25±7 | 51±12 | 52±14 | 0.263 | 0.190 | 0.967 |
| OTU00249 |  |  | Prevotella salivae | 99.05 | 9±3 | 23±4 | 30±11 | **0.023** | **0.047** | 0.967 |
| OTU00230 |  |  | Prevotella sp. oral taxon 310 | 96.85 | 7±4 | 40±13 | 43±19 | 0.074 | 0.073 | 0.967 |
| OTU00114 | Firmicutes | Gemella | Gemella morbillorum | 100 | 33±5 | 14±3 | 18±6 | **0.018** | 0.081 | 0.999 |
| OTU00225 |  |  | Gemella sanguinis | 99.38 | 60±6 | 30±5 | 50±12 | **0.003** | 0.135 | 0.967 |
| OTU00029 |  | Granulicatella | Granulicatella adiacens | 100 | 105±10 | 113±12 | 97±13 | 0.712 | 0.805 | 0.967 |
| OTU00401 |  |  | Granulicatella SRC DSC3 | 99.06 | 18±2 | 19±3 | 15±3 | 0.999 | 0.556 | 0.967 |
| OTU00072 |  | Streptococcus | Streptococcus cristatus | 100 | 22±4 | 13±4 | 20±11 | 0.081 | 0.127 | 0.967 |
| OTU00010 |  |  | Streptococcus DP009 | 99.37 | 39±6 | 12±4 | 32±12 | **0.003** | 0.178 | 0.967 |
| OTU00118 |  |  | Streptococcus mitis | 97.45 | 155±29 | 38±11 | 47±10 | **0.0005** | **0.020** | 0.967 |
| OTU00056 |  |  | Streptococcus mitis | 99.36 | 106±20 | 38±10 | 25±7 | **0.003** | **0.009** | 0.967 |
| OTU00016 |  |  | Streptococcus mitis | 98.1 | 56±17 | 4±1 | 9±3 | **0.00004** | **0.009** | 0.967 |
| OTU00022 |  |  | Streptococcus parasanguinis | 99.68 | 63±15 | 127±24 | 127±23 | 0.073 | **0.047** | 0.967 |
| OTU00213 |  |  | Streptococcus peroris | 96.5 | 18±3 | 26±6 | 28±6 | 0.695 | 0.479 | 0.967 |
| OTU00007 |  |  | Streptococcus salivarius | 99.68 | 66±17 | 77±23 | 59±16 | 0.712 | 0.790 | 0.967 |
| OTU00422 |  |  | Streptococcus salivarius | 100 | 43±9 | 86±23 | 58±12 | 0.157 | 0.556 | 0.967 |
| OTU00023 |  |  | Streptococcus sp. 11aTha1 | 100 | 116±35 | 18±3 | 31±7 | **0.00002** | **0.009** | 0.967 |
| OTU00336 |  |  | Streptococcus sp. M334 | 97.73 | 49±11 | 23±7 | 22±9 | 0.063 | 0.070 | 0.967 |
| OTU00139 |  |  | Streptococcus sp. oral strain T1-E5 | 96.81 | 41±8 | 18±3 | 21±6 | 0.081 | 0.127 | 0.967 |
| OTU00706 |  |  | Streptococcus VG051 | 98.39 | 16±3 | 22±5 | 22±9 | 0.712 | 0.443 | 0.967 |
| OTU00813 |  | Veillonella | Veillonella parvula | 99.7 | 5±1 | 36±6 | 28±7 | **0.00003** | **0.014** | 0.967 |
| OTU00068 |  |  | Veillonella sp. oral taxon 158 | 99.7 | 62±11 | 99±11 | 99±15 | **0.023** | 0.096 | 0.967 |
| OTU00045 | Fusobacteria | Fusobacterium | Fusobacterium periodonticum | 98.62 | 38±5 | 38±10 | 55±20 | 0.499 | 0.844 | 0.967 |
| OTU00011 |  | Leptotrichia | Leptotrichia genomosp. C1 | 99.35 | 19±5 | 36±13 | 20±6 | 0.999 | 0.633 | 0.999 |
| OTU00039 | Proteobacteria | Neisseria | Neisseria mucosa | 100 | 241±39 | 81±16 | 122±43 | **0.005** | 0.056 | 0.967 |
| OTU00086 |  |  | Neisseria mucosa | 99.68 | 73±32 | 16±6 | 20±10 | 0.157 | 0.605 | 0.967 |
| OTU00005 |  | Haemophilus | Haemophilus parainfluenzae | 99.35 | 98±11 | 51±11 | 54±10 | **0.013** | 0.052 | 0.967 |
| Relative abundance expressed as number of reads (mean ± s.e.m.) | | | | | | | | | | |
| P-value, Mann-Whitney's test with BH adjustment for multiple comparisons (significant values in bold) | | | | | | | | | | |

Table S5. Immunological biomarkers in the unstimulated saliva of the HC, CD and UC groups

| Biomarkers^1)^ | Observed median^2)^ | | | Median normalized by total protein^2)^ | | | P-value^5)^ | | |
| --- | --- | --- | --- | --- | --- | --- | --- | --- | --- |
|  | HC | CD | UC | HC | CD | UC | HC_CD | HC_UC | CD_UC |
| Total protein (μg/ml) | 442 (67)^4)^ | 326 (85) | 724 (131) | 1000^3)^ | 1000^3)^ | 1000^3)^ | - | - | - |
| Lysozyme (μg/ml) | 12.3 (2.7) | 2.5 (1.3) | 3.4 (4.2) | 25.5 (5.2) | 6.2 (1.6) | 3.6 (4.3) | **0.0002** | **0.007** | 0.73 |
| IgA (μg/ml) | 35  (9) | 65  (22) | 202 (59) | 88 (24) | 195 (40) | 288 (44) | **0.007** | **0.001** | **0.038** |
| LL37 (ng/ml) | 9.9 (2.7) | 14.7 (3.9) | 23.7 (7.8) | 22 (4) | 38 (9) | 35 (5) | **0.0056** | **0.028** | 0.43 |
| IL-1β (pg/ml) | 5  (34) | 41  (71) | 206 (76) | 12 (43) | 108 (110) | 298 (102) | **0.011** | **0.0017** | 0.17 |
| IL-6 (pg/ml) | 1.4 (0.8) | 3.2 (3.9) | 10.2 (1.9) | 2.7 (1.2) | 8.1 (5.6) | 11.8 (3.3) | 0.062 | **0.032** | 0.62 |
| IL-8 (pg/ml) | 63 (106) | 169 (46) | 468 (145) | 144 (149) | 499 (88) | 710 (184) | 0.085 | **0.025** | 0.19 |
| IL-10 (pg/ml) | 1.4 (0.5) | 1.3 (0.4) | 3.3 (0.5) | 1.4 (0.8) | 2.7 (1.4) | 3.7 (0.9) | 0.43 | 0.44 | 0.87 |
| IL-12(p70) (pg/ml) | 13.6 (4.4) | 19.8 (3.5) | 32.5 (4.0) | 30 (7) | 46 (15) | 40 (6) | 0.13 | 0.36 | 0.36 |
| IFN-γ (pg/ml) | 24  (7) | 23  (8) | 49  (6) | 37 (9) | 100 (30) | 51 (9) | 0.21 | 0.27 | 0.34 |
| MCP-1 (pg/ml) | 11  (18) | 28  (10) | 165 (33) | 23 (36) | 69 (26) | 211 (48) | 0.4 | **0.038** | **0.028** |
| TNF-α (pg/ml) | 3.0 (4.3) | 9.5 (2.5) | 21.5 (3.9) | 7 (6) | 26 (8) | 28 (6) | **0.035** | 0.13 | 0.92 |
| 1. Biomarkers were measured for saliva of 15 HC, 14 CD, and 10 UC subjects. | | | | | | | | | |
| 2. Observed median (left) and median normalized by total protein (middle) are shown. | | | | | | | | | |
| 3. Normalized median of total protein (middle) was set to 1000 for each group. | | | | | | | | | |
| 4. Numbers in parentheses indicate standard errors. | | | | | | | | | |
| 5. P-values (right) in bold indicate statistical significance (Mann-Whitney's test). | | | | | | | | | |

**Supplementary figure legends**

**Fig. S1. Analysis of the salivary microbiota of the HC, CD, and UC groups based on 16S data.**

(A) Principal coordinate analysis (PCoA) plot generated using unweighted UniFrac metric. The three components explained 20.84% of the variance. Blue, green, and red dots indicate healthy controls (HCs), UC, and CD samples, respectively. (B) Unweighted UniFrac distance metric (a measure of differences in bacterial community structure) between the HC and IBD (CD and UC) groups. (C) Unweighted UniFrac distance metric between the HC, CD, and UC groups. Student’s t-test was used; ^*^ P<0.01, ^**^ P<10^-5^, ^***^ P<10^-10^; mean ± s.e.m.

**Fig. S2. Weighted UniFrac distance between selected HC and IBD groups with similar and different average ages.**

HC10 indicates the subgroup of 10 healthy subjects having an average age of 25.0 years (20-32), IBD10 indicates the subgroup of IBD patients having an average age of 28.9 years (17-36), and IBD25 indicates the subgroup of 25 IBD patients having an average age of 54.6 years. Student’s t-test was used; ^*^ P<0.05, ^**^ P<0.01, ^***^ P<0.001; mean ± s.e.m.

**Fig. S3. Species abundance at the phylum level in the salivary microbiota of the HC, CD and UC groups.**

(A) Compositional proportion of bacterial species at the phylum level of the three groups. (B) Box plots of abundance comparison of major phyla between the three groups. Welch’s test with BH adjustment was used; ^*^ P<0.05, ^**^ P<0.01, ^***^ P<0.001; mean ± s.e.m.

**Fig. S4. Comparison of inflammatory biomarker levels in the saliva of the HC, CD, and UC groups.**

The average level of inflammatory biomarkers in CD and UC groups was normalized by that of HCs. The normalized values were converted to log10, and the log10 values of HCs, were set to zero. Single asterisks indicate statistical significance (P<0.05) for the HCs, and double asterisks indicate statistical significance (P<0.05) between the CD and UC samples (also see Table S5).

**Fig. S5. Relationships between relative abundance of the dominant genera and the level of immunological biomarkers in the saliva of the HC, CD, and UC groups.**

The y-axis indicates values of immunological biomarkers normalized by total protein. Linear regression was used to test for correlation. Blue, green, and red dots indicate HC, UC, and CD samples, respectively.
