# Supplementary figures and images for: Dysbiosis of Salivary Microbiota in Inflammatory Bowel Disease and Its Association With Oral Immunological Biomarkers
Source: DNA Res. 2013 Sep 7;21(1):15–25. doi: 10.1093/dnares/dst037 (PMC3925391; doi:10.1093/dnares/dst037)

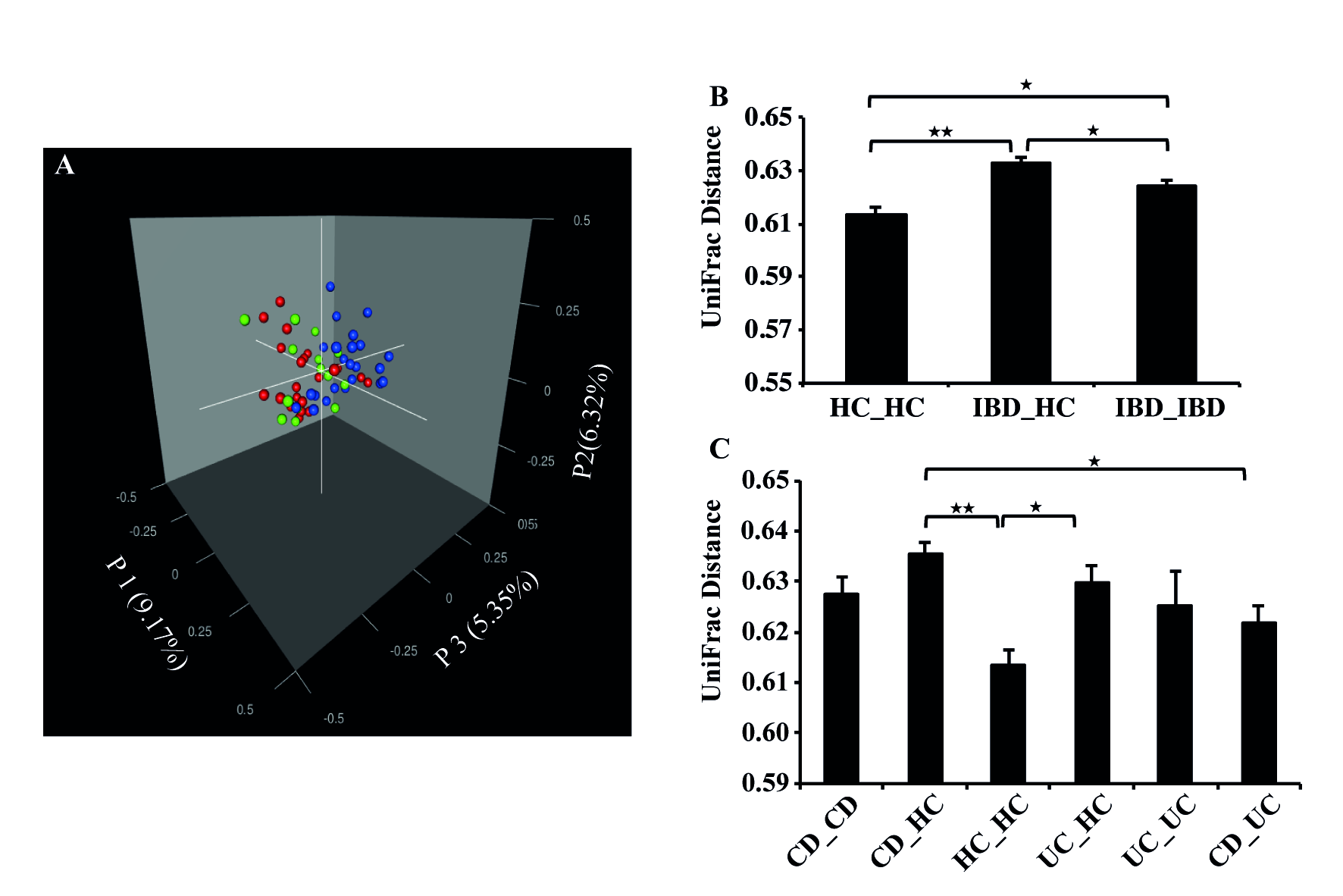

Supplement: Supplementary Data [file supp_dst037_dst037supp_fig1.tif]

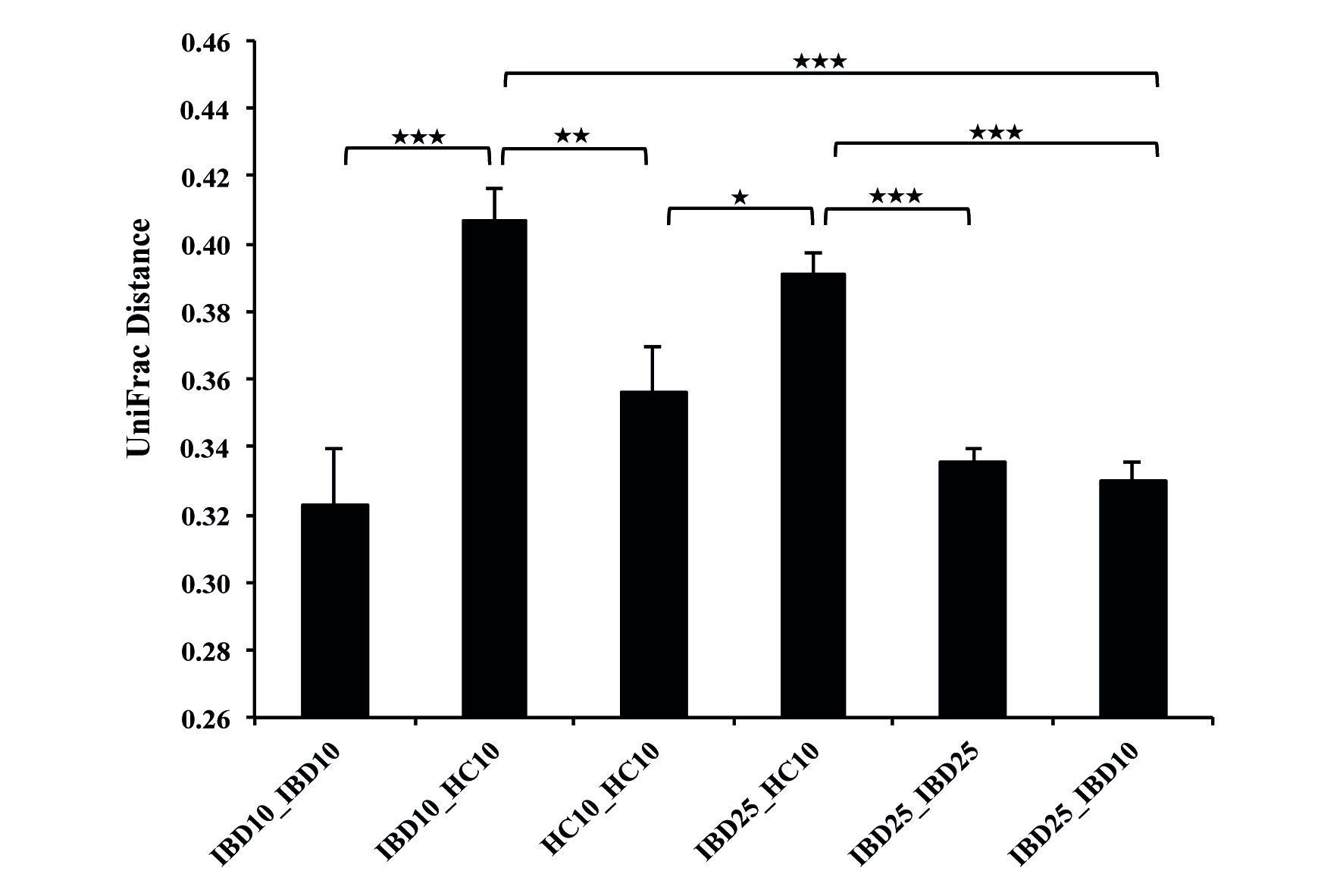

Supplement: Supplementary Data [file supp_dst037_dst037supp_fig2.tif]

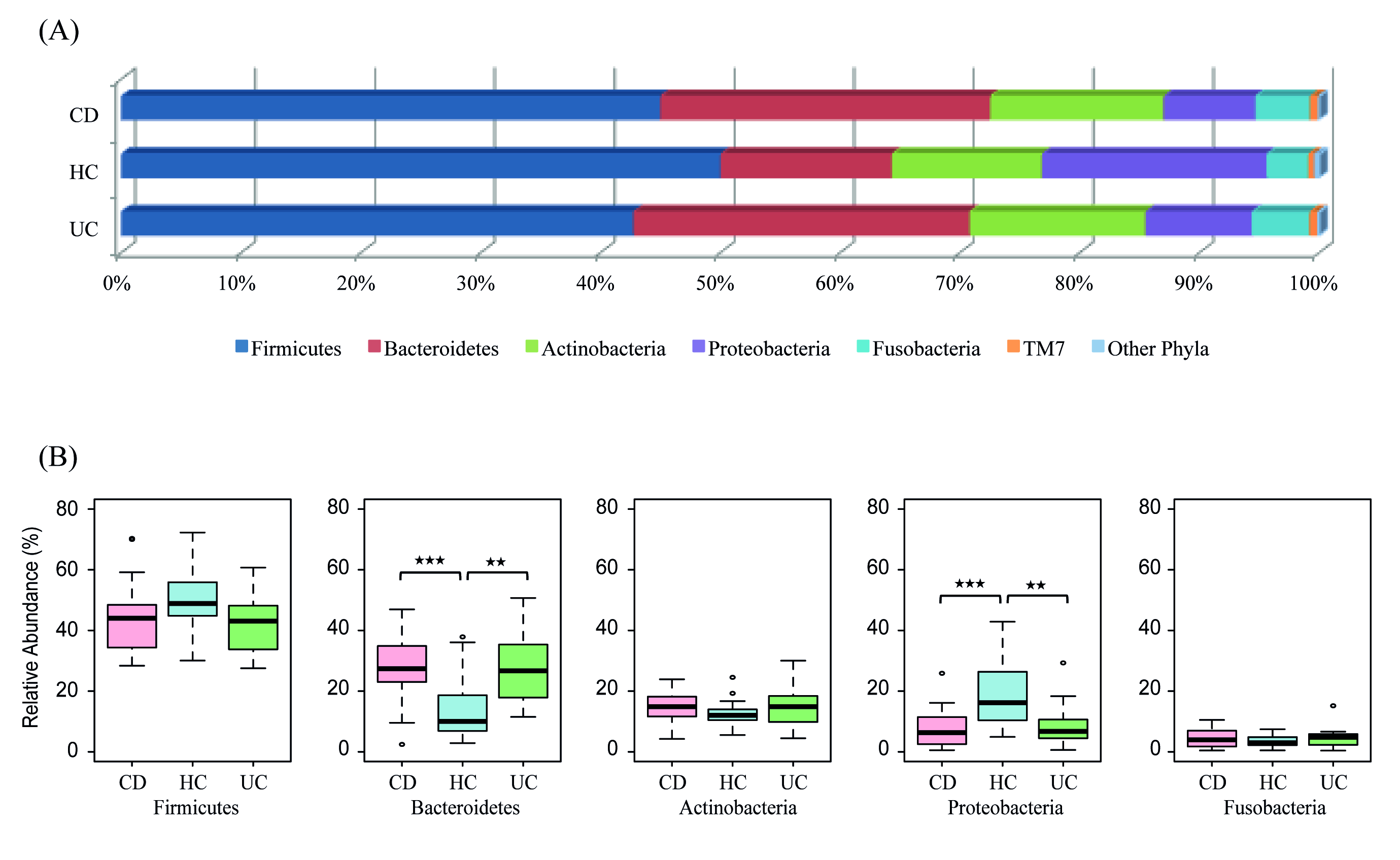

Supplement: Supplementary Data [file supp_dst037_dst037supp_fig3.tif]

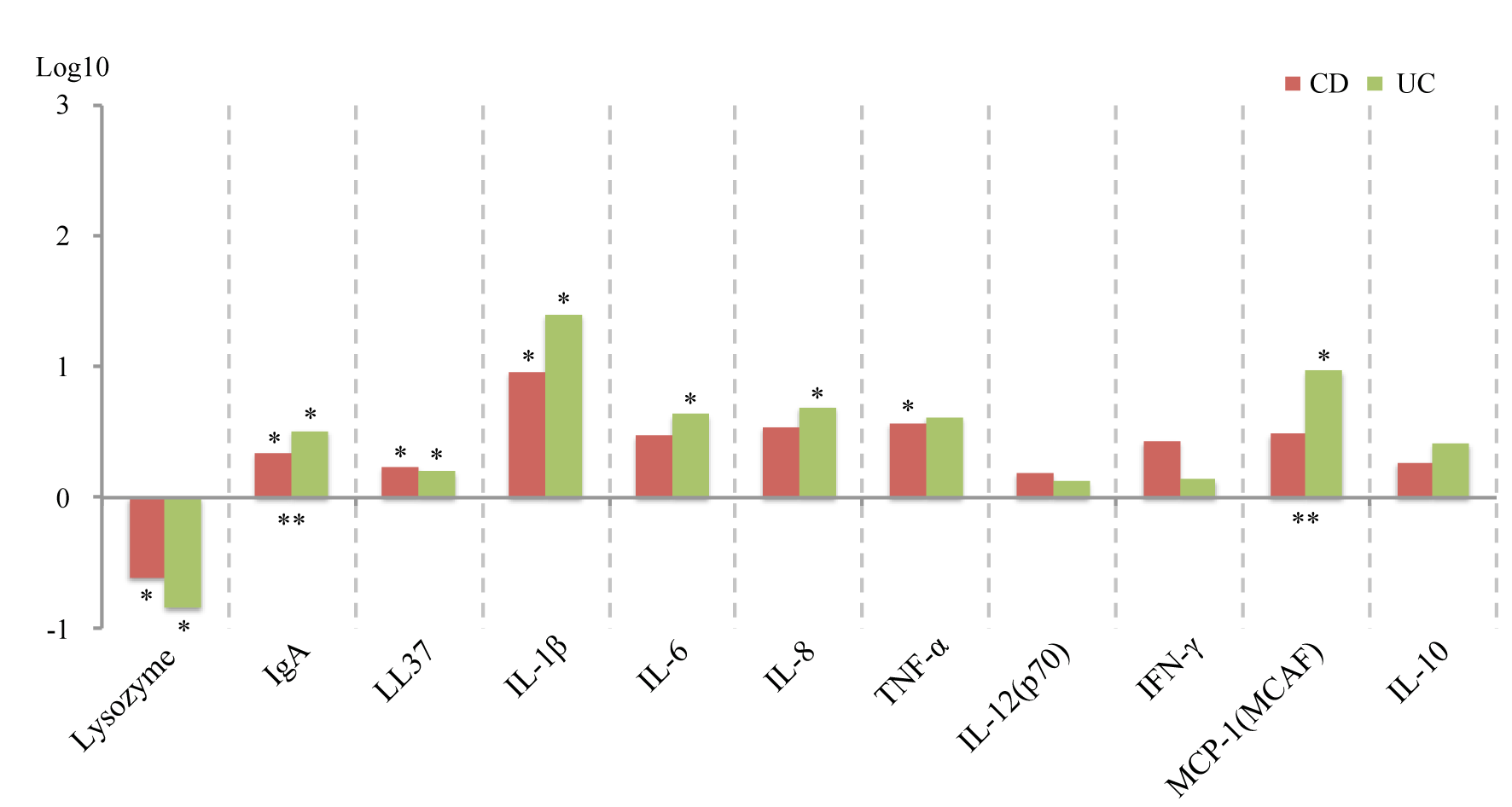

Supplement: Supplementary Data [file supp_dst037_dst037supp_fig4.tif]

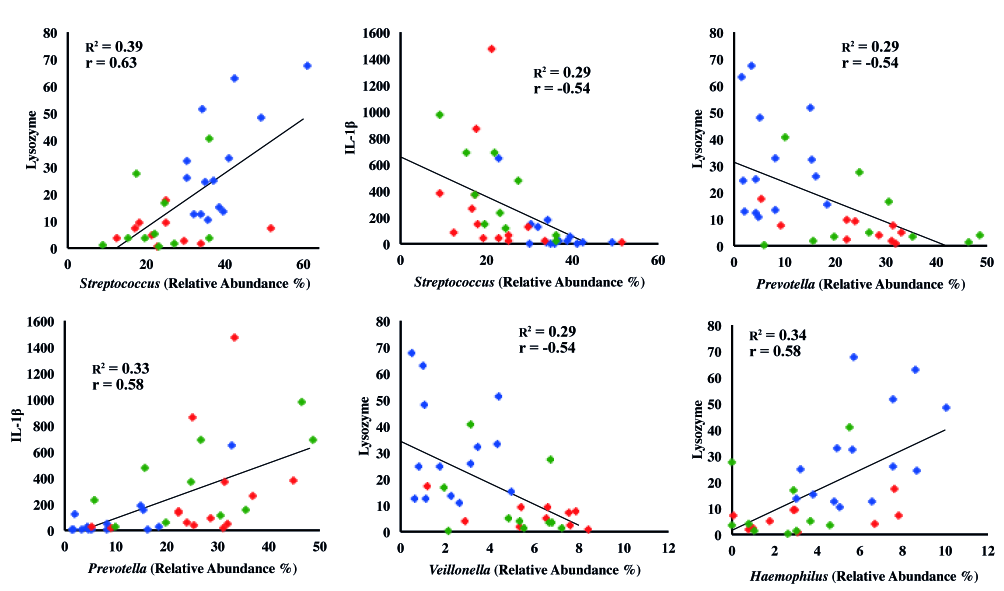

Supplement: Supplementary Data [file supp_dst037_dst037supp_fig5.tif]
